# The inhibitor of the redox activity of APE1/REF-1, APX2009, reduces the malignant phenotype of breast cancer cells

**DOI:** 10.1590/1414-431X2024e13250

**Published:** 2024-05-20

**Authors:** P.B. Siqueira, M.M.S. Rodrigues, ĺ.S.S. de Amorim, J.A. Rodrigues, M.S. Oliveira, A.S. Fonseca, B.R.B. Pires, A.L. Mencalha

**Affiliations:** 1 Departamento de Biofísica e Biometria, Instituto de Biologia Roberto Alcantara Gomes, Universidade do Estado do Rio de Janeiro, Rio de Janeiro, RJ, Brasil; 2 Laboratório de Alimentos Funcionais, Instituto de Nutrição Josué de Castro, Universidade Federal do Rio de Janeiro, Rio de Janeiro, RJ, Brasil

**Keywords:** Breast cancer, APE1/REF-1, APX2009, Migration, Invasion

## Abstract

Apurinic/apyrimidinic endonuclease 1/redox factor-1 (APE1/REF-1) is a multifunctional protein acting on cellular signaling pathways, including DNA repair and redox activities. APE1/REF-1 has emerged as a target for cancer therapy, and its role in breast cancer models would reveal new strategies for cancer therapy. APX2009 is a specific APE1/REF-1 redox inhibitor whose anticancer properties have not been described in breast cancer cells. Here, we investigated the effect of the APX2009 treatment in the breast cancer cell lines MDA-MB-231 and MCF-7. Breast cancer cell lines were cultured, and WST1 and colony formation assays were performed to evaluate cell proliferation. Annexin V-FITC/7-AAD and LDH-Glo™ assays were performed to evaluate cell death. The wound healing assay and Matrigel transwell assay were performed after APX2009 treatment to evaluate the cellular migration and invasion processes, respectively. Our findings demonstrated that APX2009 treatment decreased breast cancer cell proliferative, migratory, and invasive properties. Furthermore, it induced apoptosis in both cell lines. Our study is the first to show the effects of APX2009 treatment on apoptosis in a breast cancer cell. Therefore, this study suggested that APX2009 treatment is a promising anticancer molecule for breast cancer.

## Introduction

Breast cancer (BC) is the most common neoplasia among women worldwide (1). The late detection of BC is frequently related to increased tumor aggressiveness and metastasis. Current BC metastasis therapies are associated with severe side effects and drug resistance (2). Thus, developing and applying new drugs targeting aggressive BC cells is critical for a better outcome for patients.

Among the recently described targets for cancer therapy, APE1/REF-1 has been demonstrated to be a promissory candidate for treating metastatic disease (3-8). APE1/REF-1 is an essential enzyme in the base excision repair (BER) pathway, where it cleaves the DNA backbone 5' of the resulting apurinic/apyrimidinic (AP) site (7-10). The APE1/REF-1 redox signaling is responsible for the oxi-reduction reaction involving APE1/REF-1 cysteine 65 (C65), which maintains the target transcription factors (TFs) in a reduced state, increasing their activity (11). Recently, specific inhibitors targeting the different functions of APE1/REF-1 have been described. One of the first developed inhibitors was APX3330, a specific redox inhibitor, which was recently tested in Phase I clinical trials (NCT03375086) (12,13). APX2009 belongs to the second generation of APE1/REF-1 redox inhibitors, whose studies reported anticancer properties in treating colon, bladder, pancreatic, and prostate cancer (5-7,14).

In BC, alterations in the expression and subcellular distribution of APE1 have been observed, which have been correlated with aggressiveness and poor prognosis (15,16). The efficacy of targeting the APE1 redox domain in BC is based on its role in developing metastases. Its redox function modulates several signaling pathways implicated in the survival, proliferation, migration, invasion, angiogenesis, and metastasis of tumor cells, which are involved with TFs such as NF-κB, HIF-1α, and STAT3 (8,17). Inhibition of the APE1 redox function with APX3330 has been shown to reduce cell migration and invasion when combined with docetaxel in BC cells (17). However, the effect of APX2009 inhibitor on BC cells has not been described yet.

In the present study, we evaluated the effects of APX2009 on the aggressive feature of BC cells. We report significant findings for APX2009 as a single drug, suggesting that APX2009 treatment is a promising anticancer molecule for BC. In addition, this is the first study to show that APX2009 affected cell migration and invasion potential in a cancer cell line. Therefore, targeting the inhibition of APE1/REF-1 redox function could be a focus in the development of new anticancer drugs.

## Material and Methods

### Cell line and culture

The BC cell lines MDA-MB-231 (ATCC Cat# HTB-26) and MCF-7 (ATCC Cat# HTB-22) correspond to luminal A and basal BC subtypes, respectively (18). They were cultured in RPMI 1640 medium (Sigma-Aldrich, USA) and DMEM (Sigma-Aldrich), respectively, and supplemented with 10% fetal bovine serum (FBS) (Invitrogen, USA), 2mM L-glutamine (Invitrogen), 100 mg/mL of penicillin G, and 100U/mL of streptomycin (Invitrogen) at 37°C in a humidified atmosphere containing 5% CO_2_. Cultures were monitored for mycoplasma and certified by the DNA Diagnostic Laboratory (Brazil).

### Cell proliferation

To investigate the effect of APX2009 (Sigma-Aldrich) treatment on cell proliferation and assess the lethal and non-lethal APX2009 concentrations, we used the WST1 colorimetric assay according to the manufacturer's instructions (Roche, Switzerland). APX2009 effects were evaluated at 0.8, 4, 20, and 100 μM. The procedure consisted of seeding cells onto a 96-well plate at approximately 2,000 cells per well for MDA-MB-231 and 4,000 cells per well for MCF-7 in a volume of 150 μL of culture medium. The culture and incubation media conditions were the same as mentioned above. When cells reached 70% of confluence, the inhibitor was added to the culture medium at different concentrations. Analyses were performed at 24, 48, and 72 h of incubation. DMSO (vehicle) volumes were adjusted according to APX2009 concentration and used as control. Quantifications were performed using an ELISA reader (Polaris, USA) at 450 nm with reference at 630 nm. The number of viable cells is reported as a percentage of treated/untreated cells. The half-maximal inhibitory concentration (IC50) was calculated using GraphPad Prism 8 (USA).

### Anchorage-dependent colony formation

Anchorage-dependent colony formation assay was performed to evaluate the effects of treatment with APX2009 on cellular ability of colony formation and, consequently, cell proliferation. For this, low-density cells (4,000 cells per well for MDA-MB-231 and 8,000 cells per well for MCF-7 cells) were seeded onto 6-well plates. Four hours after seeding, the inhibitor was added to the culture medium with non-lethal APX2009 concentrations (0.8 or 4 μM) for both cell lines. After 7 days under the mentioned conditions, the cells were fixed with 100% ethanol and stained with a crystal violet solution (0.05% crystal violet in 20% ethanol). They were then washed twice with distilled water. The images were acquired using a digital camera (Samsung SM-A305GT, South Korea). ImageJ software (v.1.8.0_112, NIH, USA) was used to count the cells. Images were converted to 8-bit grayscale, and the bandpass filter was added. The color threshold was set to each image individually, and the analyzed particles with ellipses were set to a circularity between 0.60-0.80. The program counted each ellipse as a single colony.

### Cell death analysis

To evaluate whether treatment with the APX 2009 inhibitor could lead to cell death, the apoptosis assay was performed using Annexin V-FITC and 7-AAD. The cells seeded on six-well plates at 70% confluence were treated with APX2009 in increasing concentrations of 0.8, 4, 10, 20, and 50 μM to both cell lines and DMSO as vehicle control. After 24 h, cells were harvested and incubated with Annexin V-FITC and 7-AAD using an apoptosis detection kit (Thermo Fisher, USA) following the manufacturer’s instructions. Finally, cells were tested by flow cytometry (BD Accuri C6 Flow Cytometer, USA). BD CSampler Software 1.0.264.21 was used for data analysis. The data were analyzed using the percentage of cells labeled with Annexin V-FITC and 7-AAD in each quadrant.

The LDH-Glo™ Cytotoxicity Kit (Promega, USA) Assay is a bioluminescent plate-based assay for quantifying lactate dehydrogenase (LDH) release into the culture medium upon plasma membrane damage, which indicates necrosis. The cells were seeded onto 96-well plates at 70% confluence and were treated with APX2009, as cited before. LDH was used as a positive control. The bioluminescence was measured by the EnVision XCite 2105 multimode plate reader (Perkin Elmer, USA).

### Wound healing assay

The wound healing assay was performed to address the effect of APX2009 treatment on cell migration. MDA-MB-231 and MCF-7 cells were seeded onto 6-well plates and cultured as previously described. The surface of 90%-confluence cell monolayers was wounded by scratching with a 200-μL pipette tip, then incubated in a fresh culture medium supplemented with 1% FBS to prevent proliferation in the presence of APX2009 or vehicle (DMSO) at 0.8 or 4 μM for MDA-MB-231, and 4 or 20 μM for MCF-7. In this study, the migration assays were performed using concentrations of APX2009 that do not affect cell viability, avoiding indirect effects on cell migration. Cell cultures were photographed (100× magnification) at 0 and 24 h after the treatment. The analysis was performed using ImageJ software (v.1.8.0_112).

### Matrigel transwell invasion assay

The Matrigel transwell invasion assay was conducted to evaluate the effect of APX2009 treatment on the cellular invasion process. Approximately 30,000 and 100,000 cells of MDA-MB-231 and MCF-7, respectively, were seeded onto a medium containing 1% FBS with APX2009 (0.8 or 4 μM for MDA-MB-231 and 4 or 20 μM for MCF-7, non-lethal concentrations) or vehicle (DMSO) in the upper side of the transwell chamber (8 μm pore size; Corning, USA) that was coated with a Matrigel basement membrane-like matrix (1 mg/mL; BD). The lower chamber of the transwell was filled with a medium containing 10% FBS. After 24 h of incubation at 37°C and 5% CO_2_, the cells were fixed with absolute ethanol and stained with crystal violet (0.5% in 20% ethanol). The non-invaded cells present in the inserts were removed with a cotton swab. Images of the invaded cells were acquired, and five random fields were photographed (100× magnification) and counted using ImageJ software (v.1.8.0_112).

### Statistical analysis

The comparative analysis between two experimental groups was performed using the Mann-Whitney test, while the Kruskal-Wallis test, followed by Dunn's post-test, was used for multiple experimental groups. For the data with a Gaussian distribution (evaluated by the Kolmogorov-Smirnov normality test), analyses were performed with the Student *t*-test for two experimental groups and ANOVA for three or more groups, followed by the Dunnett post-test. The data are reported as means±SD. All the results with P-values <0.05 were considered statistically significant. The data analysis was performed using GraphPad Prism 8.

## Results

### The APE1/REF-1 redox inhibitor APX2009 reduced BC cell proliferation

**Figure 1:**
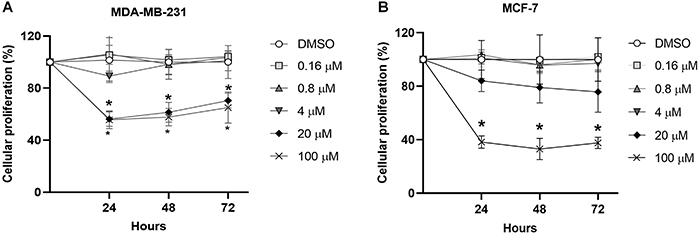
Cell proliferation assay: cellular proliferation in response to 0.16, 0.8, 4, 20, and 100 μM of APX2009 for 24, 48, and 72 h of treatment in (**A**) MDA-MB-231 and (**B**) MCF-7 breast cancer cells. DMSO was used as the respective control. The data are reported as the mean (±SD) percentages of cells in at least four individual experiments. *P<0.05, ANOVA, followed by the Dunnett post-test.

The effects of APX2009 on cell proliferation were evaluated in MDA-MB-231 and MCF-7 cell cultures. The IC50 calculated was 71 μM for MDA-MB-231 cells and 76 μM for MCF-7 cells. However, the proliferation of MDA-MB-231 cells was reduced at 20 and 100 μM of APX2009 after 24 h compared to vehicle-treated cells (Figure 1A). On the other hand, 100 μM of APX2009 reduced the proliferation of MCF-7 cells compared to the vehicle-treated cells (Figure 1B). The reduction was maintained at the 48- and 72-h evaluation times. Thus, these data suggested that APX2009 decreased MDA-MB-231 and MCF-7 cell proliferation and that MDA-MB-231 cells had a higher sensitivity.

### APX2009 treatment abrogated BC cell colony formation

**Figure 2:**
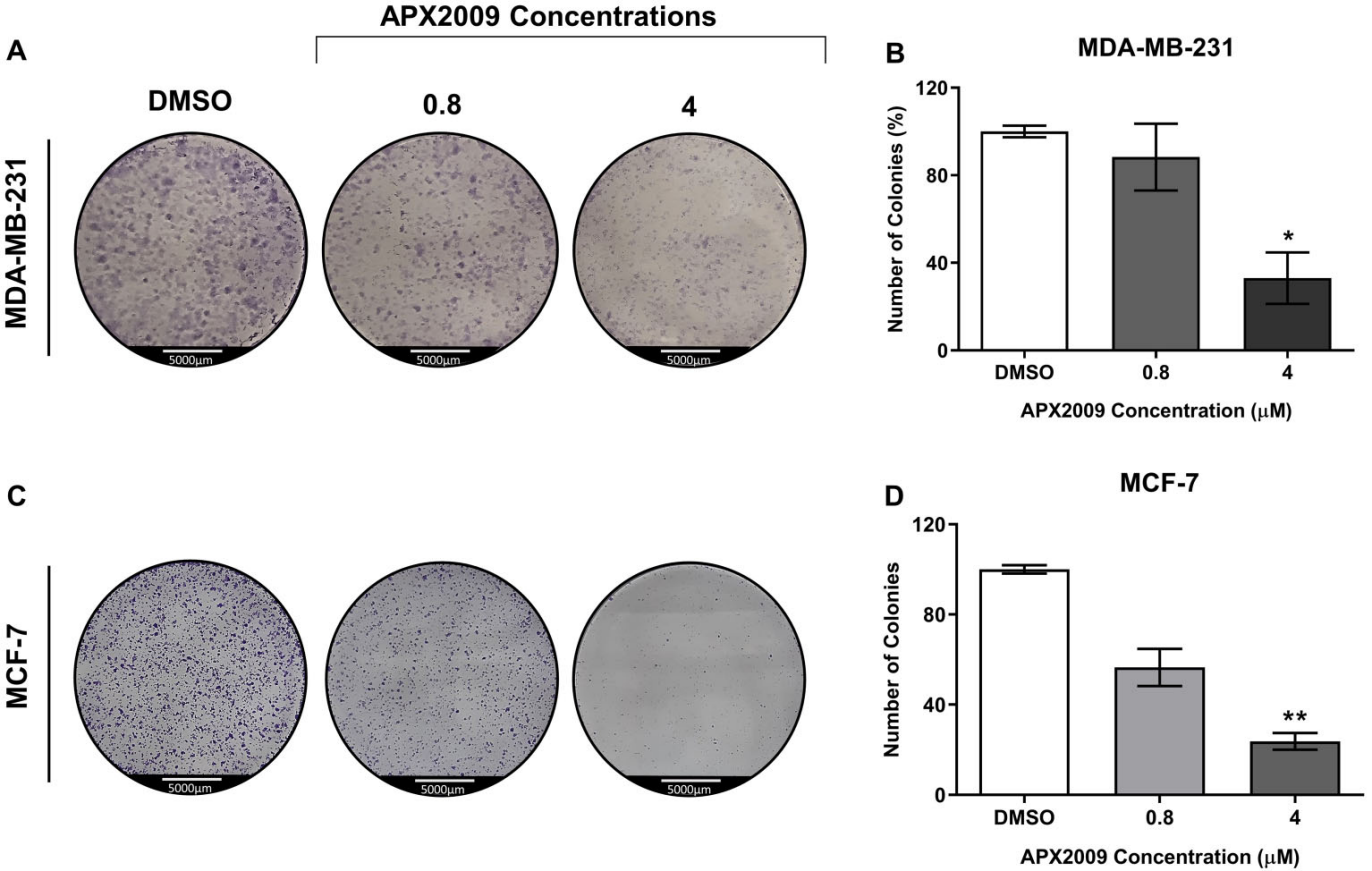
Colony formation assay: a representative colony formation assay evaluating the clonogenic ability of MDA-MB-231 cells (**A** and **B**) and MCF-7 cells (**C** and **D**) after APX2009 treatment at 0.8 and 4 µM. Data are reported as the mean number of colonies (±SD) of three individual experiments. Magnification ×100 (scale bar 5000 μm). *P<0.05, **P<0.01, ANOVA, followed by the Dunnett post-test.

To confirm the effect of APX2009 treatment on proliferation and survival, the anchorage-dependent colony formation assay was performed using 0.8 and 4 μM of APX2009 in MDA-MB-231 and MCF-7 cell cultures. Both cell lines treated with APX2009 at 4 μM significantly reduced colony formation compared to respective vehicle-treated cells (Figure 2). Thus, these results suggested that the treatment with APX2009 reduced the clonogenic potential of BC cells.

### APX2009 treatment induced BC cell death

**Figure 3:**
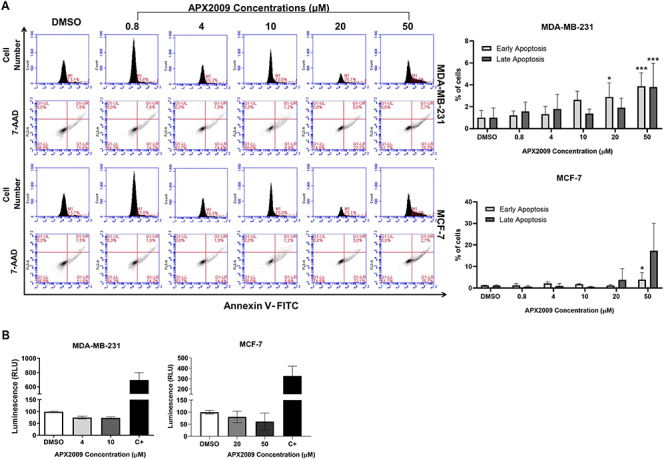
Cell death assays. After 24 h of APX2009 treatment at 0.8, 4, 10, 20, and 50 µM concentrations for both cell lines, (**A**) apoptosis was analyzed by the number of cells stained by Annexin V-FITC observed by flow cytometry analysis. Data are reported as the mean (±SD) percentages of cells of five individual experiments. *P<0.05, ***P<0.001, compared to DMSO; ANOVA. **B**, Using the LDH-Glo™ Cytotoxicity Assay, a bioluminescence plate reader measured the quantification of lactate dehydrogenase (LDH) release into the culture medium. Data are reported as the relative light unit (RLU) of at least four experiments (±SD). LDH was used as a positive control (C+).

Annexin V-FITC/7-AAD staining and quantification of LDH assay were performed to determine cell death by apoptosis and necrosis. As shown in Figure 3A (and in Supplementary Table S1), the results showed that treatment with APX2009 at concentrations of 20 and 50 µM for MDA-MB-231 and 50 µM for MCF-7 significantly increased the number of cells positive for Annexin V, indicating early stage apoptosis. In addition, treatment with the 50 µM for MDA-MB-231 showed a significant increase of late apoptosis.

Furthermore, necrosis analysis by LDH assay showed no significant differences in response to APX2009 for both MDA-MB-231 and MCF-7 cell line cultures (Figure 3B). Thus, our results suggested that the APE1 redox domain inhibitor, APX2009, led to cell death by apoptosis in both BC cell lines.

### APX2009 affected BC cell migration

**Figure 4:**
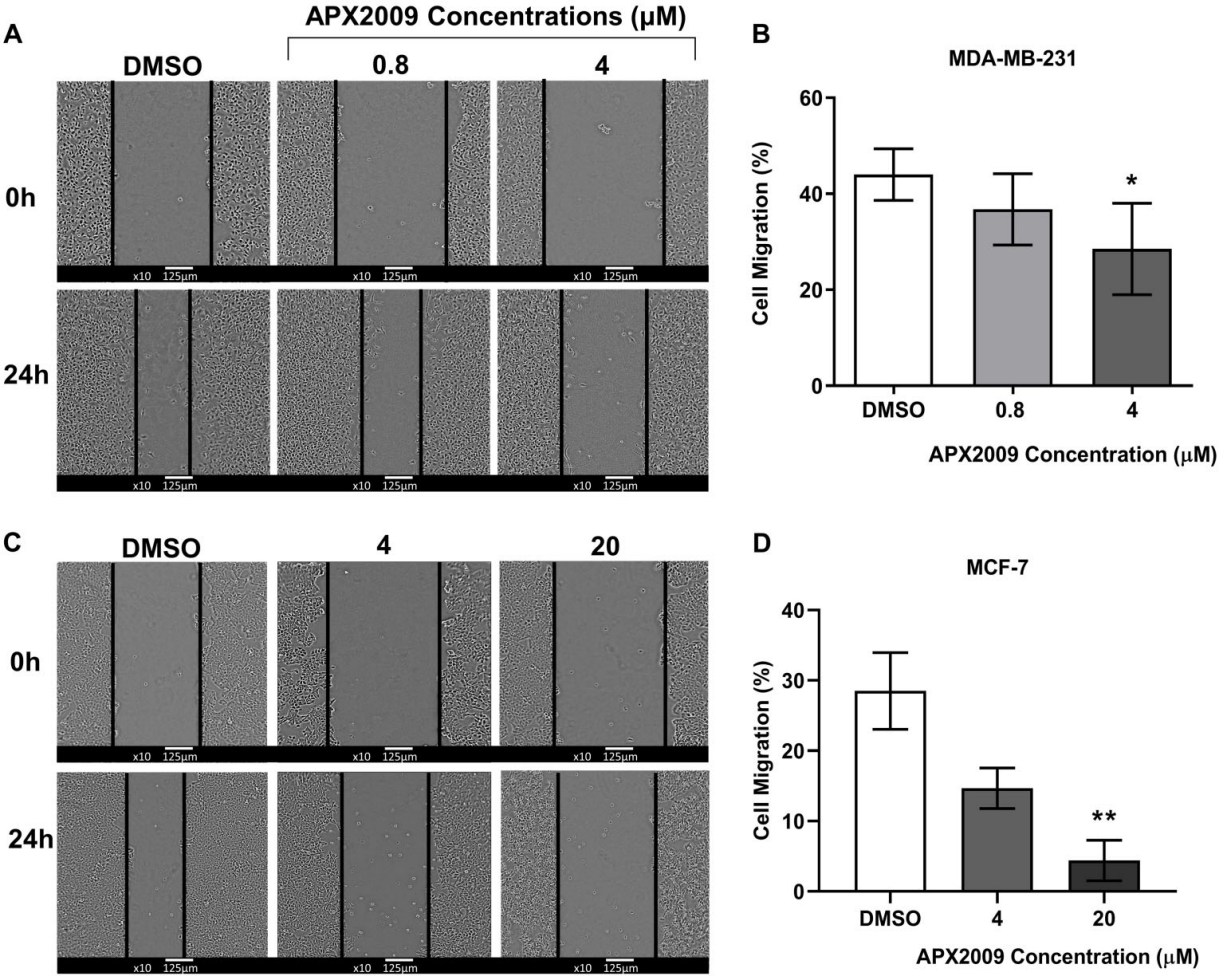
Migration assay. A representative wound healing assay evaluated cell migration 24 h after 0.8 and 4 µM APX2009 treatment of MDA-MB-231 cells (**A** and **B**) and 4 and 20 µM APX2009 treatment of MCF-7 cells (**C** and **D**). Data are reported as the mean (±SD) percentages of cells of at least four individual experiments of migratory ability as indicated by the percent of wound closure (**B** and **D**). Magnification ×100; scale bar 125 µm. *P<0.05, **P<0.01, ANOVA.

A wound-healing assay was performed to evaluate the effect of APX2009 on the motility of BC cells. The results showed that after 24 h of treatment with APX2009 at 4 μM, the migration of MDA-MB-231 cells was significantly reduced. In addition, the migration reduction was also observed in MCF-7 cells after treatment with APX2009 at 20 μM (Figure 4). Therefore, these data suggested that APX2009 efficiently reduced the migration of BC cells with non-lethal concentrations.

### APX2009 treatment decreased BC cell invasion potential

**Figure 5:**
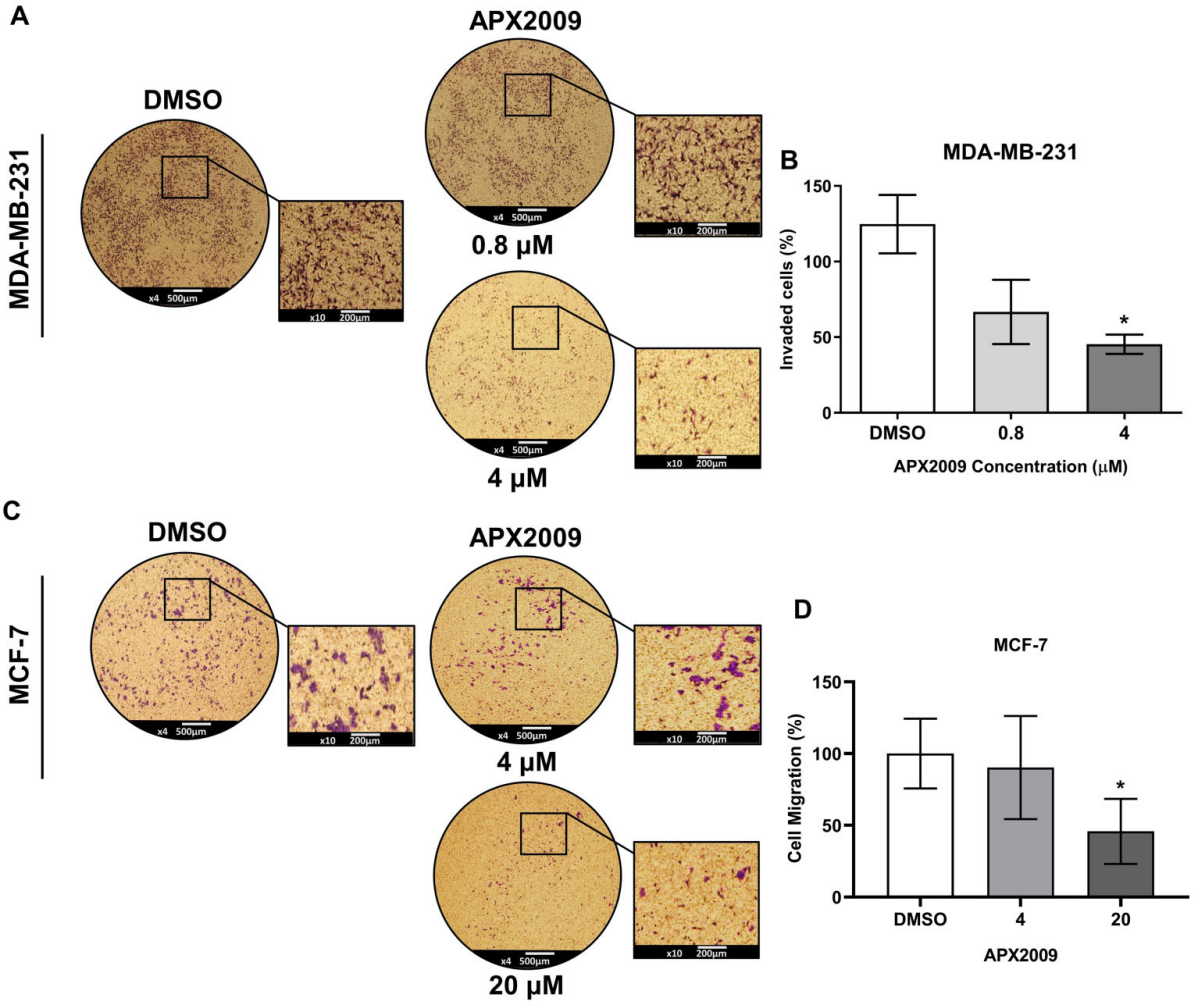
Invasiveness assay. A representative Matrigel transwell assay evaluating invasive potential 24 h after APX2009 treatment at 0.8 and 4 µM in MDA-MB-231 cells (**A** and **B**) and at 4 and 20 µM in MCF-7 cells (**C** and **D**). The cells were stained with crystal violet, and DMSO was used as the control. Magnification ×200; scale bar 500 µm, inserts 200 µm. Data are reported as means±SD of the relative invasive potential of cells in three individual experiments. *P<0.05, ANOVA.

The Matrigel transwell assay was used to evaluate the effects of the APX2009 on the invasive potential of BC cells. Reduction of cell invasion, using non-lethal concentrations, was observed at 4 μM of APX2009 in MDA-MB-231 cells (Figure 5A) and 20 μM of APX2009 in MCF-7 cells (Figure 5B). These results suggested that APX2009 influenced the invasion potential of BC cells. Together, our findings demonstrated that treatment with APX2009 reduced the proliferation and colony formation and decreased cell migration and invasion potential in BC cell lines.

## Discussion

Several authors have demonstrated the overexpression of APE1 in various tumors, such as lung, colon, ovary, prostate, and breast cancers (4-6,15-17,19,20). Therefore, APE1/REF-1 has been studied as a therapeutic cancer target due to its role in DNA repair and redox activity. Although the role of the APE1/REF-1 redox domain has yet to be thoroughly demonstrated, many transcription factors are described as targets of the APE1/REF-1 redox domain (21). Interestingly, several of these transcription factors are involved in metastasis, such as NFκB and STAT3 (22,23).

Among the existing APE1/REF-1 redox inhibitors, APX2009 harbors the second generation of molecules developed from the APX3330 molecule, extensively known as E3330 (24). To synthesize APX2009 [(2E)-2-[(3-methoxy-1,4-dioxo-1,4-dihydronaphthalen-2-yl)methylidene]-N,N-diethylpentanamide,2-[(1,4-dihydro-3-methoxy-1,4-dioxo-2 naphthalenyl) methylene]-N,N-diethyl-pentanamide], which is a naphthoquinone, Kelley et al. (24) modified the carboxylic acid moiety of the molecule APX3330/E3330 [(2E)-2-[(4,5-dimethoxy-2-methyl-3,6-dioxo-1,4-cyclohexadien-1-yl)methylene]-undecanoic acid], which is a benzoquinone, in concert with shortening the carbon chain on the double bond, whose changes modified the physical properties of the structure. They also prepared amide derivatives of the carboxylic acid, which are not charged, supporting chemical features. In addition, APX2009 has significantly shorter carbon chains on the double bond and is therefore less lipophilic than APX3330.

The phase I clinical trial of APX3330 (NCT03375086), conducted in patients with advanced solid tumors, has demonstrated a significant reduction in circulating tumor cells (CTCs) in 44% of patients after initiation of treatment. APX3330 was orally administered in 21-day cycles and tolerated at 240-600 mg daily (15,25). Phase II trials for cancer and other indications, including ocular diseases, are being developed. Considering the promising results of the NCT03375086 study and the 10 times more efficient effects of APX2009 compared to APX3330 (6,7), we understood that further studies involving the treatment of advanced solid tumors with APX2009 would shed new light on target-specific therapies. APX2009 treatments have been shown to inhibit cancer cell growth in colon, bladder, pancreatic, and prostate cancer (5-7). The role of APE1/REF-1 redox activity inhibitor APX2009 in BC cells is still unknown; thus, we investigated its effect on the malignant phenotype using the BC cells MDA-MB-231 and MCF-7 as models. Both cell lines exhibit differences in drug response by *in vitro* tests, which depend on the compound used, the subtype of cells, and the drug molecular target (26). Classically, MCF-7 presents a drug- and radio-resistance phenotype among BC cells, and it is involved in several complex mechanisms (27). Therefore, in this study, the MDA-MB-231 cells were more sensitive to the APX2009 treatment than MCF-7.

Our data showed that APX2009 treatment significantly inhibited the proliferation of BC cell lines and considerably reduced colony formation for both cell lines. Using the first-generation compound APX3330, Guerreiro et al. (17) showed a substantial reduction in colony formation in MDA-MB-231 at 50 μM of APX3330. Interestingly, our findings demonstrated that APX2009 was 12-fold more potent than APX3330 in inhibiting the proliferation of MDA-MB-231 cells compared to this previous report. In agreement, McIlwain et al. (6) showed that the APX2009 was 7.5-fold more potent in inhibiting cell proliferation in prostate cancer than the APX3330 treatment.

The treatment with the APX2009 inhibitor leads to a reduction in cell proliferation. This inhibitor's effects led to cell death at the higher concentration tested in the BC cells evaluated in this study. McIlwain et al. (6) did not observe a significant increase in cells undergoing apoptosis after treatment with APX2009 in prostate cancer cells. On the other hand, our results showed that the number of cells undergoing apoptosis significantly increased when they were treated with APX2009 at 20 and 50 µM to MDA-MB-231 cells and 50 µM to MCF-7 cells. In addition, Gampala et al. (28) showed that APX2009 treatment triggers apoptosis in malignant peripheral nerve sheath tumor (MPNST) cell lines ST88-14 and NF90-8. Furthermore, Fishel et al. (7) showed an induction of apoptosis via caspase 3/7 in bladder cancer cells after treatment with APX2009. Until now, our study is the first to show the effects of APX2009 treatment on apoptosis in BC cells.

In the development of metastases, the ability of cell migration is related to the malignant phenotype of BC cells (21,28,29). Therefore, we evaluated the effect of APX2009 on the migration of BC cells, and our results demonstrated that non-lethal concentrations of APX2009 reduced cell migration in both MDA-MB-231 and MCF-7 cells. On the other hand, Guerreiro et al. (17) showed that in MDA-MB-231 cells, treatment with APX3330, the molecule which APX2009 was developed for, significantly decreased cell migration when combined with docetaxel. However, migration was significantly reduced in other cell types, such as pancreatic cancer cells (30,31) and retinal endothelial cells (32), in response to treatment with APX3330.

The malignant phenotype of cancer cells includes the capability of tissue invasion, which is an essential step of the metastasis process (28). Regarding BC studies, MDA-MB-231 and MCF-7 cells have been frequently used as models due to their aggressive phenotype (22,33). Therefore, our study investigated the APX2009 effects on the invasion potential using both cell lines. Our results showed that treatment with APX2009 significantly reduced the invasion capability of MDA-MB-231 and MCF-7 cells. In contrast to a previous work by Guerreiro et al. (8), APX3330 alone did not significantly inhibit the invasion of MDA-MB-231 cells, whereas it was effective in combination with docetaxel. Interestingly, we found that APX2009 alone effectively reduced the invasion of these cells, which has never been described before in any other cell type. Such results showed that low concentrations of the APX2009 inhibitor significantly reduced the malignancy of these cell lines. In addition, our data showed that APX2009 treatment did not change the *APEX1* levels of the cell lines (Supplementary Figure S1), indicating a reduction in cellular events by the redox domain without changing APE1 levels in BC cells.

In summary, our findings are the first to suggest that the APE1 redox domain inhibitor APX2009 decreases the proliferation, migration, and invasiveness potential of MDA-MB-231 and MCF-7 BC cells at a low concentration. Moreover, treatment with this inhibitor at a high concentration induced apoptosis in both cell lines, revealing significant findings for APX2009 as a single drug. There is still no identification of cellular markers that were altered after treatment with the inhibitor APX2009 in BC. Therefore, large-scale studies are necessary. Our findings reinforced that targeting APE1/REF-1 by using inhibitors of its redox function could be recommended for the development of new anticancer therapies.

## Supplementary Material

Click here to view [pdf].
