# Supplementary material for: The inhibitor of the redox activity of APE1/REF-1, APX2009, reduces the malignant phenotype of breast cancer cells
Source: Braz J Med Biol Res. 2024 May 20;57:e13250. doi: 10.1590/1414-431X2024e13250 (PMC11136485; doi:10.1590/1414-431X2024e13250)
Supplement: Supplementary file 1 [file 1414-431X-bjmbr-57-e13250-suppl.pdf]

Figure S1. APEX1 expression in MDA-MB-231 (**A**) and MCF-7 (**B**) in the presence of APX2009 inhibitor. Comparison of APEX1 between cell lines (**C**). Data are reported as means±SD. Mann-Whitney test; ns: not significant.

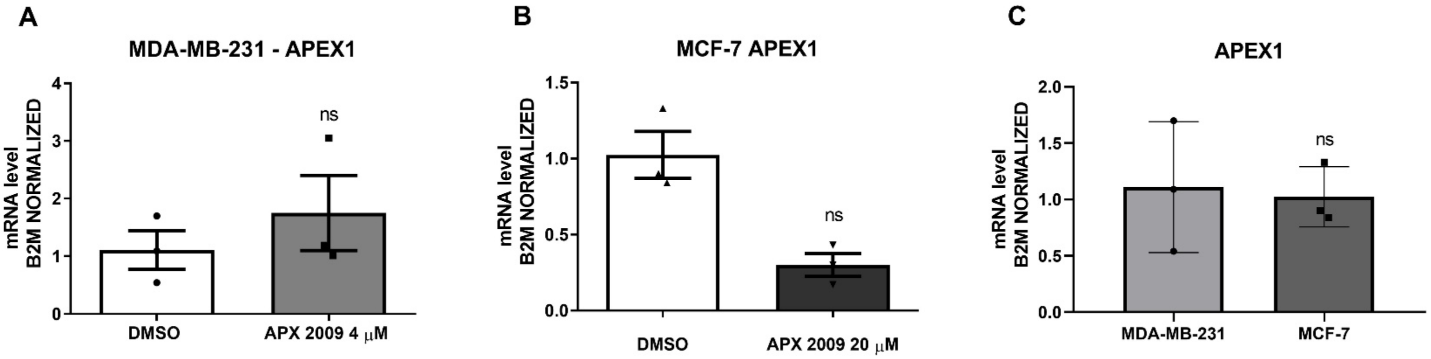

Table S1. Percentage of breast cancer cells labeled with Annexin V-FITC and/or 7-AAD.

	APX2009 concentrations (µM)											
	MDA-MB-231						MCF-7					
Quadrants	DMSO	APX 0.8	APX 4	APX 10	APX 20	APX 50	DMSO	APX 0.8	APX 4	APX 10	APX 20	APX 50
Necrosis (7-AAD+/ANNEX-)	0.00	0.00	0.00	0.00	0.00	0.00	1.04 ± 1.05	0.082 ± 0.16	0.167 ± 0.3	0.415 ± 0.8	6.168 ± 9.3	37.83 ± 45.4
Early apoptosis (7-AAD/ANNEX+)	1 ± 0.66	1.208 ± 0.41	1.323 ± 0.71	2.63 ± 0.79	2.888 ± 1.3*	3.87 ± 1.22***	1.233 ± 0.15	1.252 ± 0.83	2.139 ± 0.78	1.858 ± 0.23	1.183 ± 0.4	3.937 ± 3.2*
Late apoptosis (7-AAD+/ANNEX+)	1 ± 0.89	1.57 ± 0.86	1.794 ± 1.34	1.368 ± 0.41	1.913 ± 0.86	3.798 ± 2.17***	1.21 ± 0.23	0.7125 ± 0.53	1.023 ± 1.07	0.592 ± 0.20	3.823 ± 5.2	17.35 ± 12.73

Apoptosis was analyzed by the relative number of cells of Annexin V-FITC and/or 7-AAD staining by flow cytometry analysis. Data are reported as means±SD. *$P<0.05$, ***$P<0.001$ (Kruskal-Wallis test followed by Dunn's post-test). ANNEX: Annexin V-FITC.
